# Prehabilitation for Frail Patients Undergoing Colorectal Surgery: Lessons Learnt From a Randomised Feasibility Study

**DOI:** 10.3389/fresc.2021.650835

**Published:** 2021-05-10

**Authors:** Claire Furyk, Siva Senthuran, Dia Nye, Yik H. Ho, Anthony S. Leicht

**Affiliations:** ^1^Department of Anaesthesia, Townsville University Hospital, Townsville, QLD, Australia; ^2^Department of Anaesthesia, Geelong Hospital, Geelong, VIC, Australia; ^3^Surgical Services, Townsville University Hospital, Townsville, QLD, Australia; ^4^Department of Surgery, Townsville University Hospital, Townsville, QLD, Australia; ^5^Sport and Exercise Science, James Cook University, Townsville, QLD, Australia; ^6^Australian Institute of Tropical Health and Medicine, James Cook University, Townsville, QLD, Australia

**Keywords:** exercise, cancer, frailty, barriers, regional centre, quality of life

## Abstract

There is substantial interest by clinicians to improve the health outcomes of older and frail patients following major surgery, with prehabilitation a potential and important component of future standard patient care. We studied the feasibility of a randomised controlled trial of pre-operative prehabilitation in frail patients scheduled for colorectal surgery in regional Australia. We conducted a single blind, parallel arm, randomised controlled trial in a regional referral centre where colorectal surgical patients aged over 50 were invited to participate and screened for frailty. Frail patients were randomised to undertake either a 4-week supervised exercise program with dietary advice, or usual care. The primary outcome was 6-min-walk-distance at baseline, pre-surgery (4 weeks later) and at follow-up (4–6 weeks post-operation). Secondary outcomes included physical activity level, health-related quality of life, and post-surgical complications. Feasibility outcomes were numbers of patients reaching each stage and barriers or reasons for withdrawal. Of 106 patients eligible for screening during the 2-year study period, only five were able to be randomised, of which one alone completed the entire study to follow-up. Fewer patients than expected met the frailty criteria (23.6%), and many (22.6%) were offered surgery in a shorter timeframe than the required 4 weeks. Physical and psychological aspects of frailty and logistical issues were key for patients declining study participation and/or not complying with the intervention and/or all outcome assessments. Feasibility for a large randomised controlled trial of prehabilitation for frail colorectal patients was poor (~5%) for our regional location. Addressing barriers, examination of a large, dense population base, and utilisation of a frailty-screening tool validated in surgical patients are necessary for future studies to identify the impact of prehabilitation for frail patients.

## Introduction

Techniques in surgery and anaesthesia are continuously evolving, such that there are gradual improvements in outcomes, safety, and side-effect profiles over time. Examples of this are the development of minimally invasive procedures and fast-track programmes in colorectal cancer surgery, which have significantly reduced the surgical stress-response, the length of hospital stay and associated morbidity (1). Colorectal cancer is the third most commonly diagnosed cancer worldwide, and the fourth most common cause of cancer death (2). Age is a significant risk factor for colorectal cancer, with the majority of diagnoses in patients over the age of 60 (3). Nowadays, improvements in perioperative care have allowed major but potentially curative surgery to be offered to sections of the population who in previous generations may have been considered “too sick” or “too old”—in effect, too frail—to undergo large, invasive procedures under general anaesthesia (4).

Frailty is a clinically recognisable state of increased vulnerability to poor resolution of homeostasis after a stressor event such as surgery (5, 6). It results from aging-associated decline in reserve and function, as well as a variable burden of comorbidity across multiple physiologic systems, increasing the rate of adverse outcomes (5, 6). The prevalence of frailty in the developed world is increasing with the rate of frailty being 40–50% in patients diagnosed with colorectal cancer (7, 8). Frailty has been identified as an important risk factor for post-operative complications requiring intensive care support as evident in a study of 58,448 colectomies from the US National Surgical Quality Improvement Program database (9). In a systematic review, frail patients were generally at higher risk of complications perioperatively compared to their non-frail counterparts of the same age (10). It is now established that patients have reduced long-term survival after developing complications following major abdominal surgery, even if they survive to hospital discharge (11). Frailty is therefore an increasing clinical challenge perioperatively with ways to identify and optimise surgical recovery and beneficial outcomes in this susceptible group urgently required. Pre-operative exercise training, known as prehabilitation, is one possible method to gain these improvements.

Lower pre-operative exercise capacity and physical activity levels are reported to be independent predictors of mortality, discharge destination, and length of hospital stay for surgical patients in general (12). Frail patients are highly likely to experience lower exercise capacity and physical activity levels (6) and high rates of mortality and length of hospital stay following surgery (10). Therefore, a pre-operative focus on enhancing exercise capacity in frail patients may lead to beneficial outcomes, post-operatively. Exercise capacity can be modified through structured programs (13) with the frail elderly able to tolerate various exercise regimes (14). Further, studies of community and medical inpatients have demonstrated that multimodal interventions are feasible and beneficial in terms of reversing functional decline and improving quality of life (15, 16). Whilst studies have shown regular exercise to improve physical function with little harm during adjuvant chemotherapy for breast cancer patients (17), the evidence for prehabilitation to enhance post-operative function in other patients is less clear (18–21). A ~2.5-day shorter hospital stay was reported for frail colorectal patients following a novel trans-institutional, transdisciplinary model of care involving prehabilitation (20). A systematic review of prehabilitation studies with colorectal cancer patients aged over 60 years old reported that only 7% of the trials selected older patient groups, and frailty status was unspecified (22). The review concluded that prehabilitation was a possible strategy for enhancing physical performance pre-operatively in patients undergoing colorectal surgery, but there was no significant reduction in post-operative complications or length of hospital stay in a population aged over 60.

The type of exercise training protocols, the outcomes used and surgical case mix of the patients in prior prehabilitation studies have been diverse (18–23) with definitive benefits of prehabilitation still to be confirmed. Furthermore, the health economic benefits of pre-operative exercise training programs on patient care are unknown and encouraged (20), but if beneficial would provide an important healthcare incentive for prehabilitation to be part of standard patient care, especially for the frail elderly prior to major surgery.

This study aimed to assess the feasibility of a randomised controlled trial (RCT) of pre-operative prehabilitation in frail colorectal patients in regional Australia, including economic analysis. The feasibility study was required as this specific patient group had not previously been targeted for a RCT with an exercise intervention. Also, the planned location of regional Australia was unique, with a referral population of over 500,000 spread over an area of 80,000 km^2^. We hypothesised that compared to a control group of patients receiving usual care, frail patients undergoing elective colorectal surgery after a pre-operative tailored regimen of exercise with dietary advice would: demonstrate a greater functional walking capacity 28 days post-operatively (24); demonstrate an earlier recovery with improved post-operative 7-day physical activity levels; demonstrate improved health-related quality of life; and exhibit a reduced incidence of post-operative complications (25).

## Materials and Methods

The feasibility study was carried out in a single centre—a university-affiliated, tertiary hospital in regional Queensland, Australia, between March 2016 and November 2017. Internationally recognised guidelines for feasibility studies were followed (26) to assess the possibility of conducting a larger pragmatic study looking at both the health and economic impacts of prehabilitation in frail patients. The feasibility study processes and patient pathway were planned as for the conduction of a larger study. This was a single blind, parallel arm, RCT in frail colorectal surgical patients with the trial prospectively registered with the Australian and New Zealand Clinical Trials Registry (ACTRN12616000021471) in January 2016. Ethical approval was obtained from the Townsville Hospital and Health Service Human Research Ethics Committee (HREC/15/QTHS/176) with all patients providing written informed consent before their inclusion.

Patient inclusion criteria were as follows: patient undergoing colorectal surgery for cancer; frail or prefrail by Edmonton Frail Scale (EFS, >5 criteria) (27); able to attend exercise training in the regional city; and age ≥50. Exclusion criteria were: emergent or urgent surgery (<28 days wait); inability to speak English or documented learning impairment; and contraindications to prehabilitation based on medical comorbidities.

Patients attending colorectal outpatient clinics and booked for surgery were approached for screening and interviewed by a study nurse. Eligible consenting patients were then randomised (1:1) by computer-generated random numbers to receive either prehabilitation with dietary advice (intervention) or standard pre-operative care (control). Standard pre-operative care involved an *ad-hoc*, and as needed, program including continuation with medications and anxiety management if needed. Typically this care did not include assessment of frailty or dietary advice, unless signs of malnourishment were evident. Patients then underwent initial baseline assessments of exercise capacity *via* a 6-min walk test (6-MWT) and short physical performance battery (SPPB) (28), quality of life by EQ-5D (29), short-form 12 (30), and modified Barthel index (31), and 7-day physical activity levels using accelerometry (Sensewear Armband, BodyMedia Inc., Pittsburgh, PA). The assessments were repeated pre-operatively (~4 weeks after baseline) and at the follow-up appointment, 4–6 weeks after surgery. All assessors were blinded to group allocation.

The prehabilitation program consisted of three, 1-h sessions per week on non-consecutive days, for 4 weeks prior to surgery to increase muscular strength and cardiorespiratory/aerobic function (13). The sessions included a warm-up followed by a 30-min circuit consisting of strength and core/balance, and 20-min of aerobic exercise), and a cool down (Table 1). All sessions were fully tailored to the ability of the individual, updated as necessary, and supervised by qualified exercise physiologists. Dietary advice in accordance with Australian Dietary Guidelines (32) was provided to participants in the intervention group at the start of the 4-week program, and as needed, to ensure they could meet any increased metabolic needs.

**Table 1:** Example of a typical week of the prescribed prehabilitation program for the intervention group.

**Monday**	**Wednesday**	**Friday**
•5-min warm-up (walking)	•5-min warm-up (walking)	•5-min warm-up (walking)
•30-min of strength^a^ and core/balance^b^ circuit	•30-min of strength and core/balance circuit	•30-min of strength and core/balance circuit
•20-min of high-intensity interval walking^c^	•20-min of high-intensity interval walking	•20-min of high-intensity interval walking
•5-min cool-down (walking)	5-min cool-down (walking)	5-min cool-down (walking)

a *strength consisted of 2 x sets of 8-12 repetitions per exercise at a load of 50% predicted maximum (chest press, seated row, biceps curl, triceps extension, squat, leg curl)*.

b *core/balance consisted of single leg balance with eyes open and closed for 3-4 rounds of 1 min per round*.

c *interval walking consisted of 1 min of high intensity followed by 1-min of lower intensity (repeated)*.

The planned primary outcome for the RCT was the 6MWT distance at follow-up clinic with other data collected for secondary outcomes and economic analysis. Feasibility outcomes studied were as follows: number of patients able to be enrolled—i.e., meeting eligibility criteria for frailty screening, willing to be screened, meeting frailty criteria, and accepting randomisation; barriers to recruitment *via* open-ended questions; time required for screening and recruitment; proportion of patients enrolled and able to complete the study including all pre- and post-operative assessments, and undergoing surgery as scheduled; compliance with study protocols; reasons for patient early withdrawal; rate and type of post-operative complications, length of hospital stay, and readmission rate/reasons for frail patients; and economic analysis data collection: EQ-5D, SF-12, costs of inpatient care and complications, costs of intervention (i.e., salary, equipment, travel costs), and modified Barthel index as a measure of care dependence of patients in both groups.

Based upon historical records of annual major elective resection operations and the incidence of frailty at the tertiary hospital, and advice from colleagues at a large metropolitan hospital who care for similar patients, we expected to be able to recruit 50 patients (25 per group) over a 2-year period. Based upon this rate of recruitment, and with at least 75% of participants able to complete assessments required for the primary endpoint, a larger study involving other centres would be deemed feasible.

## Results

Recruitment to this study was slow with initial barriers identified at the 4- and 8-month time points. These barriers were: shorter than expected operative times; lower incidence of frailty than expected; patients experiencing physical and psychological effects of frailty and disease; and logistical issue associated with recruitment process and follow-up. The predominant reasons for investigator-led exclusion were that 22.6% of screened patients had surgery scheduled within 4-weeks, not allowing time for the intervention, and 23.6% fell below the EFS cut-off for frailty (Supplementary Table 1). Patient reasons for not wanting to be involved in the study were assessed through semi-structured interviews and classified into four main categories:

- Physical effects of frailty and colorectal disease: Bowel symptoms such as diarrhoea and abdominal pain, and spontaneously occurring unrelated adverse events such as falls and hospital admissions.
- Psychological effects of frailty: Patients felt that they were a burden on family and friends, especially for transport. The exhaustion component of the frailty phenotype resulted in patients being less willing to participate in an exercise intervention, in fact several visibly recoiled at the mention of the word “exercise,” and many had been advised by family members and general practitioners that they needed to rest. Conversely, several patients commented that the telephone support and extra interactions with healthcare providers as a result being in the study were invaluable.
- Timing of recruitment: Patients were approached in a busy surgical clinic immediately after receiving the news that they had colorectal cancer and required major surgery. Many patients, understandably, were minimally able to process further information, and preferred not to consider involvement in research at that time. The time required for screening and baseline assessment, ~1 h, was also a deterrent to many.
- Logistics: The ability of the frail elderly to surmount logistical problems was limited, given their reduced independence with transport and potential memory problems.

**Figure 1:**
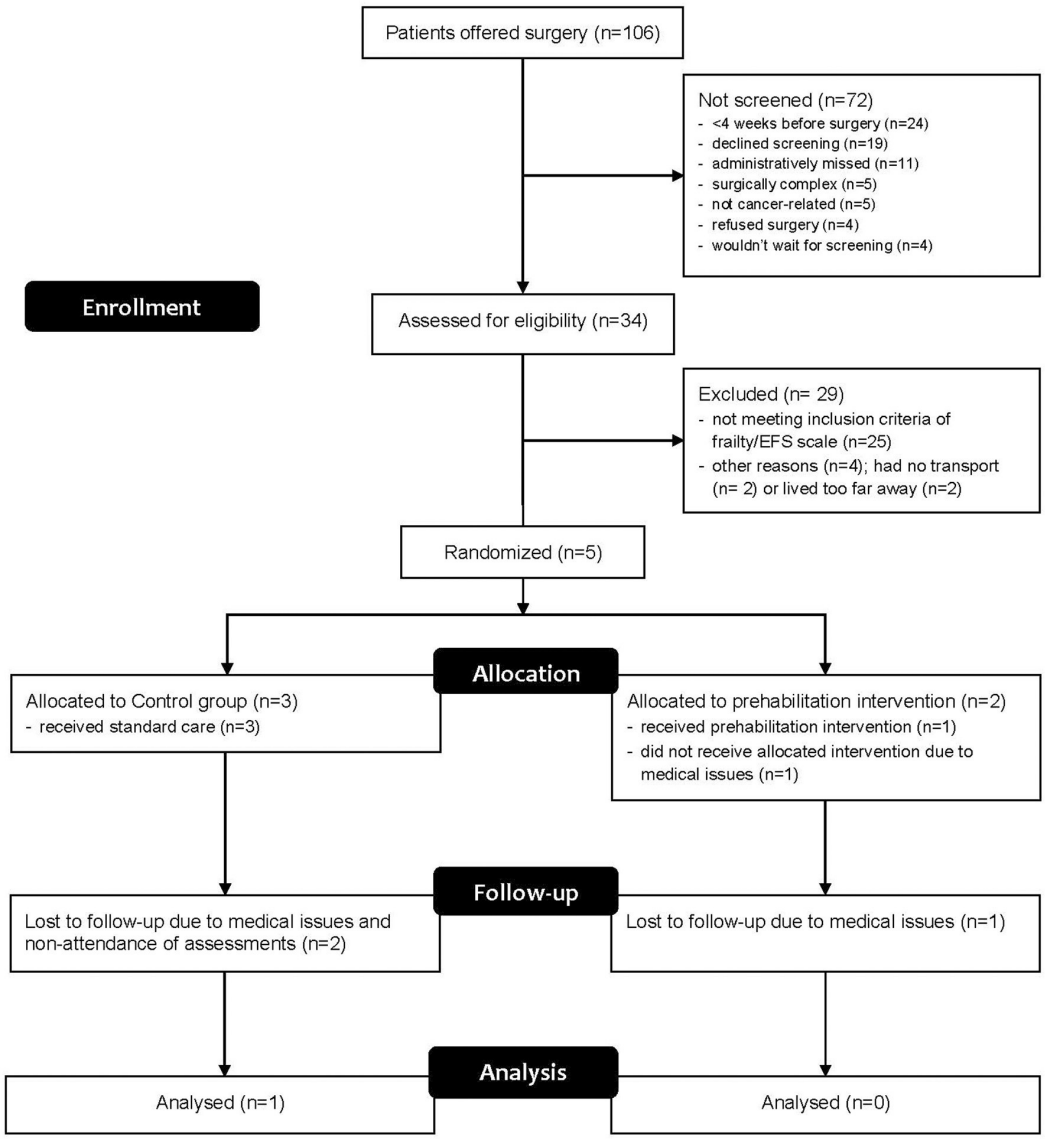
Recruitment flow diagram.

Subsequently, major amendments to the protocol were made and approved through local ethics and governance bodies, including: the inclusion of patients undergoing non-cancer colorectal surgery, such as for diverticular disease; reducing the EFS cut-off from >5 to 4, as many patients were not reaching eligibility criteria despite appearing frail to clinicians; and recruitment of exercise physiologists in surrounding towns (i.e., 100–900 km radius) to deliver the training and assessments closer to patients' residence, in order to surmount logistical issues related to travel. Despite these changes, substantial increases in patient recruitment were not achieved with similar issues raised as to those previously, and therefore the remainder of the feasibility outcomes could not be studied. The recruitment results for the feasibility study are shown in Figure 1. Only five patients out of a potential 106 were able to be randomised into the study.

Five patients were eventually recruited and one patient in the control group completed all assessments (Participant 4). All patients who undertook questionnaires and physical assessments completed these with no adverse reactions or problems. During the 6MWT, all patients walked without aids or stoppages and the distances recorded are shown in Table 2. Only one patient attended the prehabilitation sessions regularly, found them highly beneficial and was keen to continue with the program as a paying customer after surgery. The case narratives in Table 3 provide details on each participant's journey from recruitment to completion or withdrawal, giving a clear depiction of the types of issues encountered.

**Table 2:** Exercise capacity *via* 6-min walk test distance for participants at each assessment time point.

**Participant**	**Group**	**Baseline** ** (m)**	**Pre-operative** ** (m)**	**Follow-up** ** (m)**
1	Intervention	NA	NA	NA
2	Control	451	460	NA
3	Control	325	NA	NA
4	Control	380	440	415
5	Intervention	472	488	NA
Mean ± SD		407 ± 58	463 ± 20	415

*NA, not assessed; m, metres*.

**Table 3:** Participant narratives.

Participant 1	60-year-old man with chronic diverticulitis, scoring five on the frail scale mainly for mood, weight loss and hospital admissions. He was given a date 4 weeks away for bowel resection and was distressed by the wait. He was randomised to the treatment arm but was bedbound by ongoing abdominal pain. He was diagnosed with chronic myelomonocytic anaemia, splenomegaly, and a splenic bleed. He never managed to attend for baseline assessments and was withdrawn from the study.
Participant 2	73-year-old visibly frail man after chemoradiotherapy treatment for rectal carcinoma scoring seven on the frail scale. Initially not recruited due to a postural drop in blood pressure but returned after medication adjustment and was keen to be recruited. He was randomised to the control group, but before attending baseline assessments was admitted to hospital after collapsing at home. He then decided to use herbal treatment instead of undergoing surgery, and was withdrawn from the study. He was subsequently involved in a motor vehicle crash suffering multiple fractures and a closed head injury, necessitating a 4-week hospital admission.
Participant 3	82-year-old man with bowel cancer, scoring seven on the frail scale after a recent admission with anaemia for blood transfusion and weight loss. He was keen to participate, was enrolled and randomised to the control group. He attended baseline assessments but had difficulty remembering to fill in the activity diary and found the accelerometer uncomfortable. He was nearly impossible to contact by phone but needed frequent reminders to attend appointments and to return accelerometers by post. His operation went ahead but post-operatively he was admitted several times to his local hospital with vomiting and diarrhoea and lost 16 kg in 4 weeks. He was withdrawn for failure to attend assessments or return the accelerometer.
Participant 4	64-year-old female who was working full time and caring for two grandchildren, but scored four on the frail scale for mixing up the clock face and medication. She was randomised to the control group, and managed to complete all the assessments, but forgot to document exercise training in the diary.
Participant 5	57-year-old female with chronic diverticulitis scheduled for bowel resection, scored four on the frail scale for having minimal social support and subjectively poor health. She was randomised to the intervention, completed all the pre-operative assessments and undertook 5 out of 12 training sessions with the other 7 sessions prevented due to abdominal symptoms. She enjoyed and felt benefit from the training and was keen to continue after surgery. Unfortunately, she suffered a severe anaphylactic reaction to chlorhexidine at the commencement of her surgery, and the operation was abandoned and rescheduled, so she was withdrawn from the study.

## Discussion

The current study demonstrated poor feasibility of a RCT for pre-operative prehabilitation in frail colorectal patients within regional Australia. Over 2 years, only ~5% of eligible patients were willing to participate with significant barriers to participation identified. Consideration of barriers, population base, and frailty-screening tools for surgical patients are crucial to confirm the impact of prehabilitation for frail, elderly, colorectal patients.

There has been much interest in the past few years concerning frail patients and post-operative outcomes (33), with studies comparing frailty scores as outcome markers (8–10). Several studies examining prehabilitation in elderly colorectal patients have been conducted in metropolitan centres in Canada (19, 23, 34), the United Kingdom (35), Hungary (21), and a regional hospital in the Netherlands (12). These studies have demonstrated improved post-operative functional walking capacity (19, 21), and return to baseline exercise capacity after neoadjuvant chemoradiotherapy (35). Chia and colleagues (20) highlighted the potential of prehabilitation for frail colorectal patients with a ~2.5-day shorter hospital stay following a novel trans-institutional, transdisciplinary model of care. In contrast, no improvements were observed for duration of hospital stay or for post-operative mortality and morbidity following a trimodal (i.e., physical, emotional, and nutritional) prehabilitation program in non-frail colorectal patients (21). Whilst frailty may pose a significant confounding influence on patient outcomes, prehabilitation of frail patients may be associated with better outcomes from surgery (20, 36). Therefore, the current study aimed to expand upon these prior findings of frail colorectal patients (20) with a RCT within regional Australia however, significant barriers to recruitment were encountered. Notably, patients were scheduled for surgery within a short timeframe, due to both treatment urgency as well as efficiency drives to ensure a full operating schedule within the regional public health system (i.e., surgery brought forward to replace a cancelled surgery). The length of the prehabilitation program in the current study was in accordance with the average waiting period for colorectal surgery within the public health sector of regional Australia. Further, it aligned with other prehabilitation programs (33) and/or longer than other prehabilitation programs for abdominal surgery (37). The optimal length of prehabilitation has not been confirmed with recent reviews highlighting the large variability in prehabilitation programs for cancer and frail patients (33, 38). Therefore, it is generally accepted that surgery for treatment of cancer is offered with as minimal a delay as possible, in order to prevent progression of the disease. However, there is retrospective evidence that a delay of up to 12 weeks does not adversely affect outcomes for up to 5 years (39). Further, therapeutic delay (>35 days) led to similar overall or cancer-free survival in patients with primary colorectal cancer who underwent curative surgical treatments (40). Therefore, a dedicated commitment to prehabilitation (e.g., 2–12 weeks) as pre-surgical care may be fundamental to optimise patients' functional status, along with enhancement of many other risk factors such as nutritional status, psychological wellness, diabetes control, iron stores, and smoking status (21, 41, 42). Future studies comparing multimodal prehabilitation with a delay, vs. benefits of earlier surgery without prehabilitation, and identifying cohorts of patients who benefit from each strategy would be of great interest. While surgery timelines were a significant limitation, many patients in the current study were unwilling or unable to participate due to the physical and psychological effects of frailty and/or cancer (6, 43). Therefore, multidisciplinary approaches focusing on holistic care are necessary to support patients at this vulnerable time. The range of issues experienced by patients and encountered by clinicians in the current study pose significant challenges to research and future therapies to enhance surgical outcomes and long-term healthcare of these patients. Addressing these important barriers is vital for future robust RCT evaluations of prehabilitation in this vulnerable population (43).

Despite the aforementioned barriers, frail patients were able to complete exercise capacity assessments and undertake prehabilitation in accordance with recommended exercise guidelines (13). Recently, a small number of frail, older patients with colorectal cancer (*n* = 14) were able to complete an at-home, digital prehabilitation program of exercise and nutrition (20). Collectively, our and prior (20, 44) results highlighted that frailty alone is not a contraindication to prehabilitation but frail colorectal patients require additional support to engage with prehabilitation programs, as described above. Importantly, frailty needs to be identified initially by clinicians. A recent systematic review (33) highlighted that frailty should be assessed in surgical patients with an appropriate instrument. We elected to use the EFS as our tool to identify frailty as it has been the most validated in clinical and research use to date (27). However, the scale has been validated in medical patients rather than a surgical population and therefore we found that a significant proportion of our final cohort of patients (73.5%), although subjectively quite frail, did not score highly enough to be eligible for inclusion (Supplementary Table 1). Thus, tool selection is of paramount importance when classifying frailty, as one needs to identify a cohort of patients who are frail enough to have the potential for significant gains from increased exercise capacity, but not so frail that they are unable to be studied. In the current study, a large proportion of the screened patients were not frail enough by this tool to be included that may represent a bias. Those patients exhibiting greater frailty may be more likely to not consider enrolment in a prehabilitation program due to the physical and psychological effects of frailty and/or cancer (6, 43). Therefore, addressing the identified barriers at diagnosis may be crucial to engage patients with prehabilitation for benefits in this vulnerable population (43). For example, engaging family of the patient in all discussions, addressing expectations of patients in terms of effort needed and benefits of exercise to health, engaging medical/nursing staff to champion the program, and logistical issues such as transport and regular communication (43).

A range of tools to identify the clinical syndrome of frailty have been reported and validated over the last several years (27, 45, 46). They can be scored using objective measures, subjective questioning or mixed methods. Ideally they cover physical, psychological, and social domains as all three contribute to the frailty syndrome (6). Examples of mixed scores in widespread use are Fried's Phenotype of Frailty or the Frailty Index developed by Mitnitski (45), as well as the EFS (27). The Risk Analysis Index (47, 48) is a subjective frailty screening tool shown to have improved mortality outcomes when used as a trigger for senior pre-operative consultation in a large cohort of mixed surgical patients in a US Veteran Affairs hospital. The Risk Analysis Index could be a more appropriate choice of tool to identify frailty in a surgical cohort. Another approach to identify frailty could be based on objective markers of frailty such as muscular weakness and sarcopenia (46, 49, 50). Handgrip strength was reported to correlate negatively with overall frailty (50) while psoas muscle area was positively correlated with outcomes after major surgery (46, 49). Both of these musculoskeletal assessments are relatively simple and quick to assess however, only represent physical aspects of frailty. Therefore, the most appropriate frailty tool remains to be determined to assist clinicians with therapeutic options including prehabilitation.

As recently highlighted (33), there exists very little evidence for the use of prehabilitation for frail populations (i.e., five studies) with more RCT studies needed. Most studies to date have been conducted in metropolitan areas that likely can support the design of RCT studies. The proportion of colorectal cancer patients eligible and willing to travel to participate in our regional research study was as expected (~10% once eligibility modifications were in place) however, the absolute number was very small. Therefore, a larger, dense population base is warranted for future studies. Based upon our results, studies aiming to recruit 100 patients in a year should recruit from services that perform at least 1,000 colorectal surgeries annually. Also, as only one participant out of five managed to complete all the assessments to follow-up in our study, loss to follow-up rates may be high (up to 80%) and therefore may require an even larger population base. Future, large-scale studies of pre-operative prehabilitation in frail colorectal patients, possibly across multiple sites and/or metropolitan centres, are needed to confirm the health and economic benefits of prehabilitation for this patient group. A more pragmatic quality improvement initiative, such as prehabilitation embedded into routine care (20), may provide a valuable design for future studies to assist clinicians and patients with optimising healthcare delivery and outcomes. Further, the use of remote care (*via* technology) may support prehabilitation as routine care in the future. Recently, the use of a wearable (smartwatch/phone application) over 2-weeks was reported to increase physical activity levels and functional (aerobic) fitness of colorectal cancer patients (37). Incorporation of wearables and/or telehealth may support patients to undertake prehabilitation by overcoming issues identified in the current study (e.g., transport logistics, comfort, etc.). Future studies are encouraged to clarify the role of remote care to aid prehabilitation outcomes, especially in remote settings.

Finally, the current study highlighted the value of conducting a pilot study, especially for a vulnerable group of patients. Despite all good intentions to undertake a novel and important intervention within a regional setting, significant issues were encountered in this feasibility study that would limit a larger scale trial. Such learnings have provided clear direction and economic savings for the conduct of future trials in this topic by clinicians.

## Conclusions

Improving surgical outcomes for high-risk, frail patients is a key health goal and prehabilitation interventions merit robust assessment in this group with the best patient-centred approach yet to be determined. Addressing barriers, examination of a large, dense population base and utilisation of a frailty-screening tool validated in surgical patients are necessary for future studies to clarify the impact of prehabilitation and those patients who can benefit most. Finally, a pilot or feasibility study can provide clinicians with valuable guidance when studying future “real-life” interventions for frail populations.

## Data Availability Statement

The original contributions presented in the study are included in the article/Supplementary Material, further inquiries can be directed to the corresponding author.

## Ethics Statement

The studies involving human participants were reviewed and approved by Townsville Hospital and Health Service Human Research Ethics Committee (HREC/15/QTHS/176). The patients/participants provided their written informed consent to participate in this study. Written informed consent was obtained from the individual(s) for the publication of any potentially identifiable images or data included in this article.

## Author Contributions

CF, SS, YH, and AL: conceptualisation. DN and AL: data collection. CF, SS, DN, YH, and AL: data analysis/interpretation and manuscript revisions. CF, SS, and AL: manuscript writing. All authors contributed to the article and approved the submitted version.

## Conflict of Interest

The authors declare that the research was conducted in the absence of any commercial or financial relationships that could be construed as a potential conflict of interest.
