# Supplementary material for: Prehabilitation for Frail Patients Undergoing Colorectal Surgery: Lessons Learnt From a Randomised Feasibility Study
Source: Front Rehabil Sci. 2021 May 10;2:650835. doi: 10.3389/fresc.2021.650835 (PMC9397917; doi:10.3389/fresc.2021.650835)
Supplement: Supplementary file 1 [file Table_1.docx]

# Supplementary Table 1. Edmonton Frail Scale (EFS) score for all 34 screened patients.

| EFS score | Number of patients (%) |
| --- | --- |
| 0 | 5 (14.7) |
| 1 | 6 (17.6) |
| 2 | 6 (17.6) |
| 3 | 4 (11.8) |
| 4 | 4 (11.8) |
| 5 | 5 (14.7) |
| 6 | 1 (0.03) |
| 7 | 2 (0.06) |
| 8 | - |
| 9 | - |
| 10 | - |
| 11 | 1 (0.03) |
